# *In situ* preparation, electrical and surface analytical characterization of pentacene thin film transistors

**DOI:** 10.1063/1.4895992

**Published:** 2014-09-21

**Authors:** R. Lassnig, B. Striedinger, M. Hollerer, A. Fian, B. Stadlober, A. Winkler

**Affiliations:** 1 Institute of Solid State Physics, Graz University of Technology, Petersgasse 16, A-8010 Graz, Austria; 2 Materials Division, Joanneum Research Materials, Franz-Pichler-Straße 30, A-8160 Weiz, Austria

## Abstract

The fabrication of organic thin film transistors with highly reproducible characteristics presents a very challenging task. We have prepared and analyzed model pentacene thin film transistors under ultra-high vacuum conditions, employing surface analytical tools and methods. Intentionally contaminating the gold contacts and SiO_2_ channel area with carbon through repeated adsorption, dissociation, and desorption of pentacene proved to be very advantageous in the creation of devices with stable and reproducible parameters. We mainly focused on the device properties, such as mobility and threshold voltage, as a function of film morphology and preparation temperature. At 300 K, pentacene displays Stranski-Krastanov growth, whereas at 200 K fine-grained, layer-like film growth takes place, which predominantly influences the threshold voltage. Temperature dependent mobility measurements demonstrate good agreement with the established multiple trapping and release model, which in turn indicates a predominant concentration of shallow traps in the crystal grains and at the oxide-semiconductor interface. Mobility and threshold voltage measurements as a function of coverage reveal that up to four full monolayers contribute to the overall charge transport. A significant influence on the effective mobility also stems from the access resistance at the gold contact-semiconductor interface, which is again strongly influenced by the temperature dependent, characteristic film growth mode.

## I. INTRODUCTION

Electronic devices based on organic semiconductors are on the verge of taking over large shares of several markets currently dominated by inorganic systems. The widespread scientific interest in organic electronic devices can be largely attributed to the inherent possibility to deposit and pattern the involved semiconducting materials at room temperature. This enables the creation of low-cost, large-area electronic functions on flexible substrates via comparably inexpensive processes and therefore to realize applications not feasible with silicon or other inorganic transistor technologies.^1-4^ While the possibility to create and optimize organic devices is evident to the present date, many of the underlying principles affecting critical device parameters such as performance and lifetime are not yet fully understood and controllable, as outlined in a number of review articles.^5-8^ In general, the film morphology of the active layer, the molecular ordering, and chemical impurities are recognized as prominent factors for the attainable organic thin-film transistor (OTFT) performance.^9,10^ Additionally, high performance organics are often sensitive to oxygen,^11,12^ humidity,^13^ and light exposure,^14^ leading to sometimes severe device degradation under ambient conditions and limited operational lifetime.

One of the most promising and most frequently studied organic semiconductors for transistor application is pentacene (C_22_H_14_), mainly due to the observed high field effect mobilities of greater than 1 cm^2^/Vs.^15^ Typically, pentacene is deposited in vacuum on silicon dioxide as a gate dielectric with gold electrodes in a bottom or top contact configuration. Subsequently, the devices are electrically characterized in air. However, recently some *in situ* device fabrication and electrical characterization have been reported as well. Kiguchi *et al*.^16^ were one of the first to study the conductivity of pentacene films *in situ* continuously during film growth, and found that the accumulation layer is just a few nm thick. Similar experiments were performed by Liu *et al*.,^17^ who additionally focused on the role of pentacene purification^18^ and on the influence of vacuum breaking on the device performance.^19^ Furthermore, *in situ* and real time electrical measurements were also carried out in the group of Biscarini,^20,21^ who studied the influence of the deposition rate on the device performance and on the number of active layers contributing to the drain current. Finally, the evolution of mobility, threshold voltage, and hysteresis during pentacene deposition was investigated *in situ* by Fiebig *et al*.^22^

In order to go beyond this type of *in situ* research on organic semiconductors, we present the analysis of the semiconducting layer in organic field effect transistors through a unique combination of *in situ* layer deposition, real-time electrical *and* surface analytical characterization, with all investigations being performed under ultra-high vacuum conditions. With a special sample holder, which allows cooling and heating of the sample between 120 K and 800 K during electrical measurements, we can realize unprecedented investigations during deposition and during layer thinning by thermal desorption. Auger electron spectroscopy (AES) is applied to control the chemical composition of the surfaces involved prior and after film deposition. The deposited film can be desorbed and analyzed by Thermal Desorption Spectroscopy (TDS) in a controlled way, thus allowing to establish the thermal stability of the film. Due to the fact that repeated film deposition and removal can be realized without breaking the vacuum, we are able to test the influence of various parameters on the reproducibility of the transistor characteristics. Argon ion sputtering for sample cleaning can be performed throughout the stages of the device fabrication. However, in this work we focused on the realization of highly reproducible devices, which was achieved by creating a saturation carbon layer, both on the gold contacts and the gate oxide surfaces. In order to demonstrate the performance and versatility of our experimental setup, we have investigated the classic OTFT model system pentacene on silicon dioxide with bottom-contact gold electrodes.

## II. EXPERIMENTAL SETUP

### A. Sample preparation and mounting

**FIG. 1:**
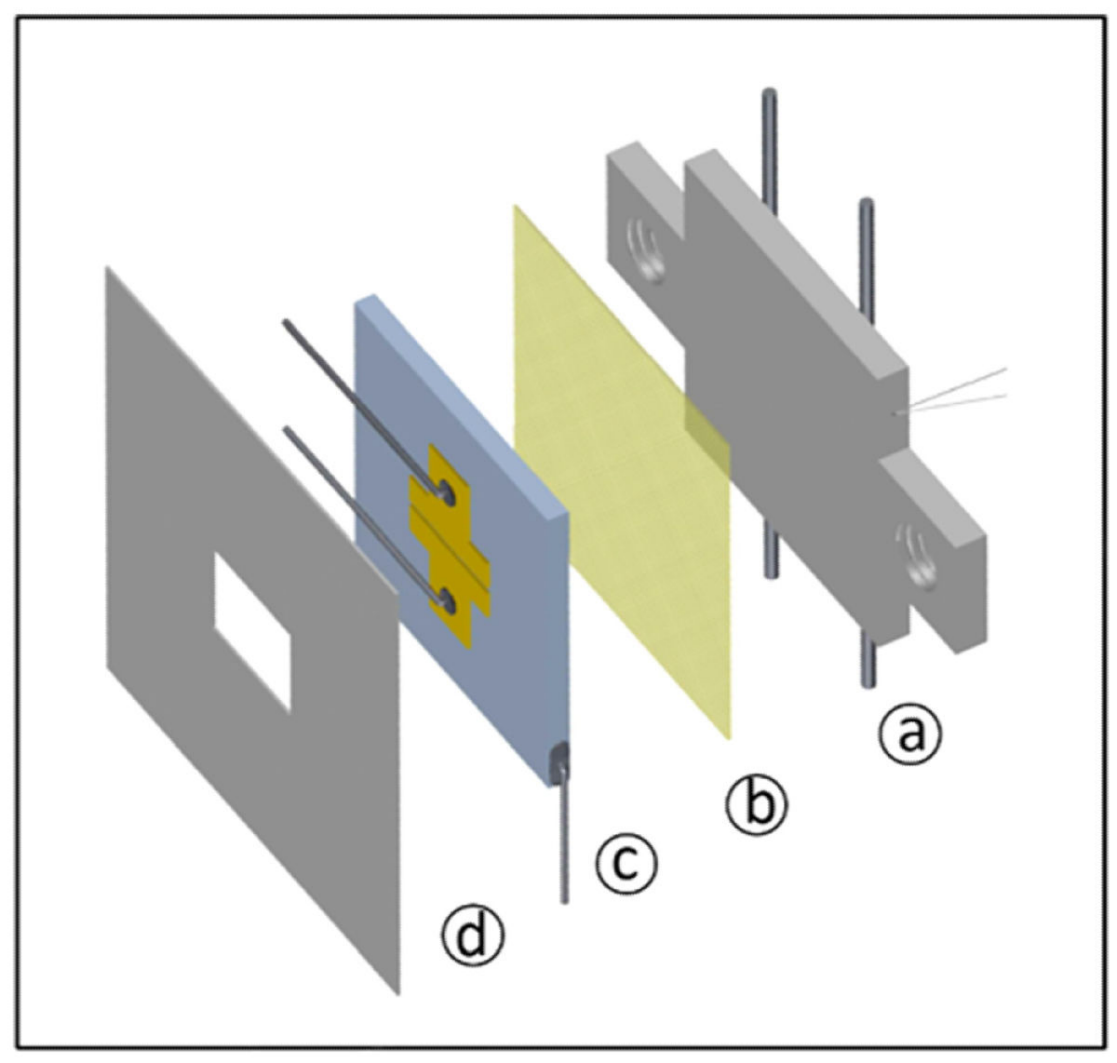
Exploded assembly of sample and sample holder. (a) Stainless steel plate with spot-welded Ta-wires for heating and Ni-NiCr thermocouple, (b) insulating mica foil, (c) Si/SiO_2_ sample device with Au-contact pads and Ni contacting wires, and (d) Al-shadow mask.

The standard samples consist of highly p-doped silicon wafer pieces (0.6 mm × 1.0 cm × 1.0 cm) with a bulk resistivity of <0.01 Ω cm and a 150 ± 10 nm thick dry-oxide layer on top, manufactured by Siegert Wafer.^23^ Following an oxygen plasma etch cleaning step, 60 nm thick gold contacts were thermally deposited through a shadow mask, forming a 25 *μ*m × 4 mm channel area. Contacting of the source and drain gold electrodes was established by connecting thin nickel wires (0.1 mm diameter) with conductive silver solder to the Au electrodes and by employing the sample side of the highly conducting silicon as gate contact (Fig. 1). The back ends of the nickel wires connect to Kapton insulated, electrically shielded cables leading to the outside of the ultra-high vacuum chamber via BNC-feedthroughs. The sample mounting itself is based on attaching the 1 cm^2^ silicon wafer onto a stainless steel back plate, with a thin mica sheet in between for electrical insulation. The steel plate (1 mm thickness), which serves as the sample carrier, can be heated resistively and/or cooled with liquid nitrogen through two 0.25 mm diameter tantalum wires. A thermocouple, spot-welded to the steel plate’s backside, monitors the temperature in the accessible range from about 120 K to 800 K. Ceramic washers between the sample and its mounting screws, in addition to the mica sheet, keep the sample insulated from the steel plate, which allows electrical measurements during resistive heating of the sample. The cooling is performed by filling the L-shaped sample holder with liquid nitrogen. In front of the sample, a thin aluminum shadow mask with a rectangular aperture (3 mm × 4 mm) is positioned, which limits the area of pentacene deposition, and hence reduces the leakage and off-currents.

### B. Vacuum setup and surface analytical instrumentation

The ultra-high vacuum chamber is pumped by a rotary vane roughing pump and two turbo molecular pumps. A base pressure of 2 × 10^−8^ mbar can be reached without bake-out of the vacuum system. Sample cleaning prior to semiconductor deposition and modifications on finished devices can be performed by Argon ion sputtering (Specs IQE 11/35 with direct gas supply). Typically, mild sputtering with ion energies of 800 eV is applied. The main purpose of sputtering is the removal of the most significant residual impurity in the form of carbon. Carbon is always present on the sample surface (both on the silicon dioxide channel surface and the Au electrode surfaces) after it had been kept in air for a significant time, or after thermal desorption induced hydrocarbon dissociation. Actually, in this work we intentionally abstained from intensive sputtering, but rather tried to prepare stable and reproducible gold and silicon dioxide surfaces covered by a monolayer (ML) of carbon. Such stable surfaces were produced by repeated deposition and thermal desorption of pentacene, as checked by AES.

Furthermore, the experimental setup includes a quadrupole mass spectrometer (QMS) with a measurement range of 0–200 amu, used for residual gas analysis and thermal desorption spectroscopy. For TDS measurements, the sample is positioned in front of the QMS and the mass spectrometer is set to a specific mass, which is then monitored as a function of the sample temperature; in our case, we used a heating rate of 1 K/s. From the obtained desorption spectra, one can attain information on the chemical composition, coverage, and thermal stability of the analyzed film. Since the mass of pentacene (278 amu) is beyond our maximum measurement range, we used the cracking mass of 125 amu for the desorption analysis, which turned out to be one of the most prominent cracking masses in our mass spectrum. By multiplexing the QMS other masses, if required, can be simultaneously measured.

The semiconductor deposition system consists of a resistively heated stainless steel Knudsen cell, a double shutter system, and a quartz microbalance (QMB). The Knudsen cell is placed in a larger metal tube with openings for simultaneous deposition onto the sample and on the oscillating quartz crystal. The pentacene used for our experiments was purchased from Tokyo Chemical Industries (TCI) purified twice in a train sublimation system. The quartz microbalance (resonance frequency *f* ≈ 6 MHz) is water cooled throughout deposition to minimize temperature induced effects and is positioned close to the sample. From the sensitivity of the quartz (*S* = 2.26 × 10^−6^cm^2^s/g)^24^ and the density of pentacene (*ρ* = 1.3 g/cm^3^), we obtain the correlation between the frequency change Δ*f* and the mean thickness *d* of pentacene (according to *d* = *S*^−1^Δ*f*/(*f*^2^*ρ*)) of 1 Hz ≡ 0.094 nm. However, calibration of sub-monolayer films via atomic force microscopy (AFM) revealed that actually 1 Hz ≡ 0.08 nm, due to the actual geometry of the deposition setup. In combination with the nominal thickness of about 1.6 nm for a monolayer of standing pentacene molecules,^25^ a change of 20 Hz in the QMB resonance frequency corresponds to about 1 ML. In addition, the double shutter system allows selective deposition onto the QMB and/or the sample. The combination of the shutter system with the established pentacene sticking coefficient of s = 1 (see below) enables a very precise control of the deposited layer thickness, as well as an accurate deposition rate and exposure time control. Typically, we used quite low deposition rates of about 0.16 nm/min (0.1 ML/min).

The chemical composition of the gold electrode surface and the silicon dioxide surface was checked by Auger electron spectroscopy, using a cylindrical mirror analyzer from Staib Instruments. Since the diameter of the electron beam estimates to about 0.5 mm, a representative SiO_2_ surface area outside the 25 *μ*m wide channel was analyzed to monitor the chemical composition in the channel area. An *ex situ* atomic force microscope (NanoSurf Easyscan2) was used to characterize the morphology of the pentacene films. For electrical characterization, a Keithley 2612A double source meter unit (SMU) was used, which was addressed via a specifically designed LabView^®^ program.

## III. RESULTS AND DISCUSSION

### A. Initial surface preparation and characterization

The model device samples used in this work had a bottom gate-bottom contact configuration, where the gold contacts were deposited by evaporation after an oxygen plasma cleaning step in a plasma preparation chamber. Thereafter, the samples were exposed to air and installed, without further cleaning, onto the appropriate sample holder for electrical contact and transferred into the UHV chamber for surface analytical and electrical characterization. On such a freshly installed sample, the gold contacts and the silicon dioxide surfaces were contaminated with an ill-defined carbon layer. Moderate sputtering with Argon ions (800 eV, 10 min at 8 × 10^−6^ mbar Ar) is sufficient to remove the carbon from the surface. Heating of the sputtered surface to 800 K does not lead to further contamination. However, deposition of several monolayers of pentacene on the cleaned surface and subsequent desorption leads to some pentacene decomposition, resulting again in a carbon contamination.

**FIG. 2:**
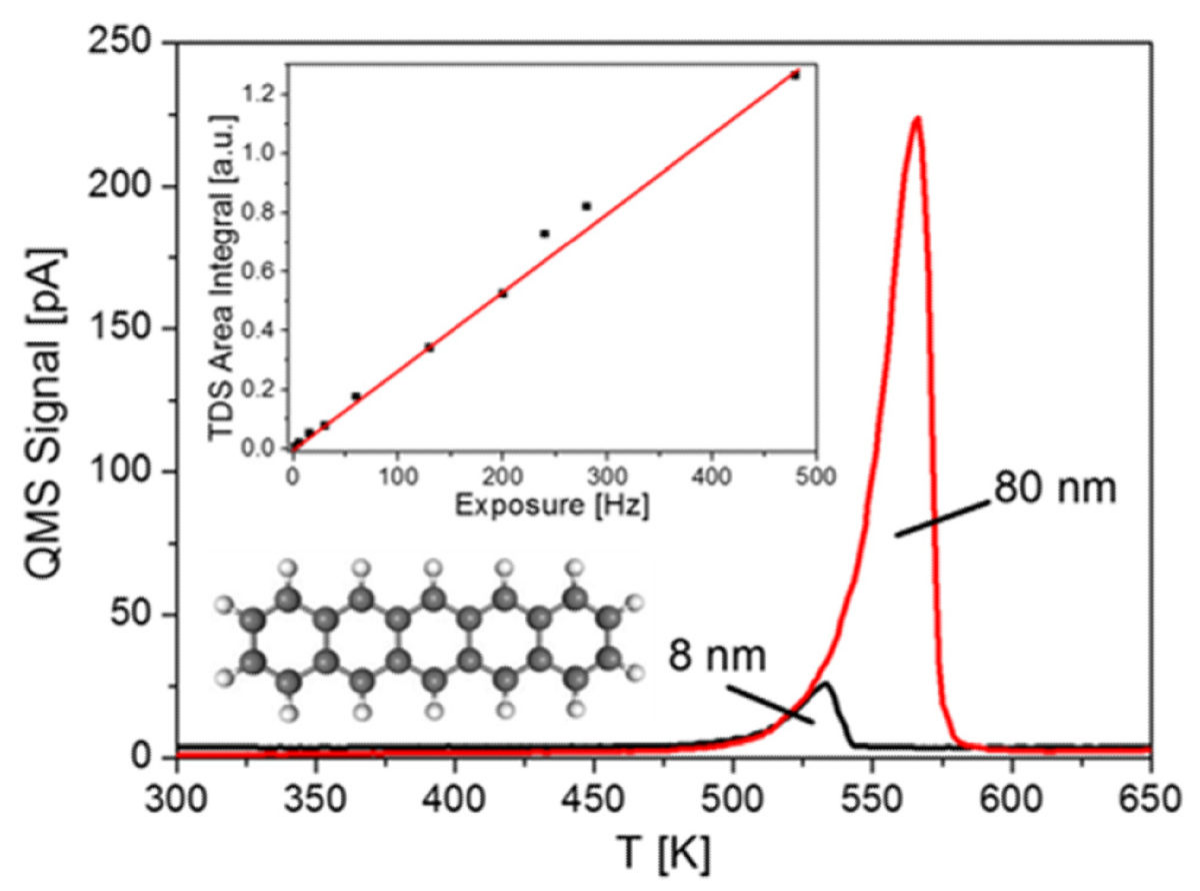
Desorption spectra of pentacene from the Au-SiO_2_ surface after deposition of 8 nm and 80 nm thick films at 300 K, respectively. Heating rate *β* = 1 K/s. Regarding the meaning of the measured temperature see the supplementary material.^27^ In the insets, the molecular structure of pentacene and the correlation between TDS area and pentacene exposure are shown.

Desorption spectra for pentacene after depositing 8 nm and 80 nm, respectively, which are characteristic for zero-order desorption, i.e., multilayer desorption,^26^ are shown in Fig. 2. The temperature scale as shown in this figure does not accurately reflect the true desorption temperature, due to the existence of a considerable temperature difference between the stainless steel sample holder, where the temperature is measured, and the sample surface, attributable to bad thermal conductivity of the insulating mica foil and the silicon sample itself. However, a simple first-order temperature correction is possible just by comparing the peak maximum of pentacene desorption from the sample with that from the stainless steel sample holder, which the thermocouple is connected to. It turns out that a measured peak temperature of 530 K for an 8 nm thick pentacene layer corresponds to a true desorption temperature of 420 K (see supplementary material).^27^ This agrees nicely with the literature data.^28^ It should be noted that for layers of this thickness (multilayers) no difference in the desorption behavior from the gold and silicon dioxide surface can be observed. Repeated pentacene adsorption and desorption eventually leads to a saturation coverage of carbon, which is largely inert against further decomposition of pentacene. Such a surface is very stable and allows repeated fabrication of transistors with high reproducibility. Unfortunately, based on the experimental techniques used (Auger spectroscopy) we cannot comment on the specific chemical nature of the carbon layer. However, from the literature it is known that carbon covered silicon dioxide shows a higher hydrophobicity.^29^ On the contrary, transistors fabricated on freshly installed samples without further surface treatment show quite strong variations in their electrical performance. In the inset of Fig. 2, the correlation between the integrated TDS areas and the deposited amount, as measured by the quartz microbalance, is plotted. The linear relationship demonstrates the constant sticking coefficient in the whole coverage range. This suggests that the sticking coefficient of pentacene on the carbon covered gold and silicon oxide surface is one, independent of the coverage, allowing a precise determination of the deposited pentacene amount.

### B. Electrical characterization of a stable pentacene device

**FIG. 3:**
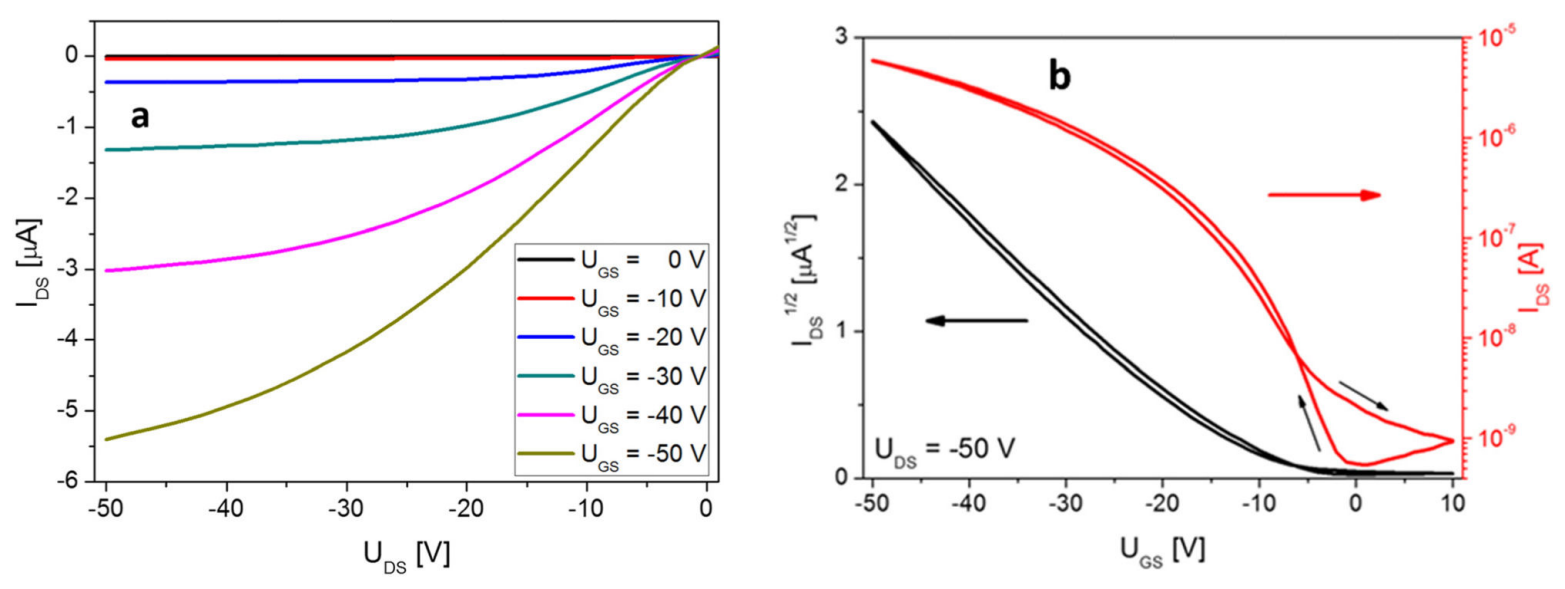
Output (a) and transfer characteristics (b), measured at 300 K, of an 8 nm thick pentacene film after deposition on a carbon covered device at 300 K.

The deposition of pentacene on the carbon saturated gold and SiO_2_ surfaces yields highly reproducible device properties. In Fig. 3, representative output (a) and transfer characteristics (b) for an 8 nm thick pentacene device are presented, with the film being deposited and measured *in situ* at 300 K. The transfer curve is shown in forward and reverse measurement and is indicating an almost hysteresis free behavior. For the analysis of the obtained output and transfer curves, we use the formalism for standard MOSFET devices:^30^

(1) $$I_{\mathrm{DSLin}}=C_{G}\mu_{\mathrm{Lin}}\frac{W}{L}\left[(U_{\mathrm{GS}}-U_{T})U_{\mathrm{DS}}-U_{\mathrm{DS}}^{2}∕2\right]\;\text{for}\;|U_{\mathrm{GS}}-U_{T}|>|U_{\mathrm{DS}}|(\text{linear regime}),$$

(2) $$I_{\mathrm{DSSat}}=C_{G}\mu_{\mathrm{Sat}}\frac{W}{2L}{\left[U_{\mathrm{GS}}-U_{T}\right]}^{2}\;\text{for}\;|U_{\mathrm{DS}}|>|U_{\mathrm{GS}}-U_{T}|>0(\text{saturation regime}).$$

These equations describe the dependence of the drain current *I*_DS_ from the drain-source voltage *U_DS_* and the gate voltage *U_GS_* in the various regimes, with the parameters gate dielectric capacitance *C_G_*, carrier mobility in the semiconductor *μ*, channel width *W*, and channel length *L*. In our particular case, *W*=4 mm, *L*=25 *μ*m, and *C_G_*=23 nF/cm^2^ (150 nm thick SiO_2_ with ε(SiO_2_)=3.9). The scan rate for the output and transfer curves is 5 V/s. From Figs. 3(a) and 3(b), we obtain field effect mobilities **μ*_Sat_*=2.3 × 10^−3^ cm^2^/Vs, **μ*_Lin_*=1.4×10^−3^ cm^2^/Vs, a threshold voltage of *U_T_* =−13 V, an onset voltage *U*_on_=−1 V, and the ratio *I*_on_/*I*_off_≈ 1 × 10^4^. **μ*_sat_* was derived from the slope of the linear part of the $\sqrt{I_{\mathrm{DS}}}$ vs *U_GS_* plot, with *U_DS_*=−50 V and *U_GSmax_*=−50 V. The threshold voltage *U_T_* is then given by the intercept of this slope with the abscissa. The linear mobility *μ*_Lin_ was derived from the linear part of the transfer curve for *U_DS_*=−3 V (not shown). The onset voltage *U_on_* marks the gate-source voltage at which the drain current reaches a minimum, and is derived from the log(*I_DS_*) vs *U_GS_* plot. The inverse of this slope is called sub-threshold swing and amounts to 3.6 V/decade.

**FIG. 4:**
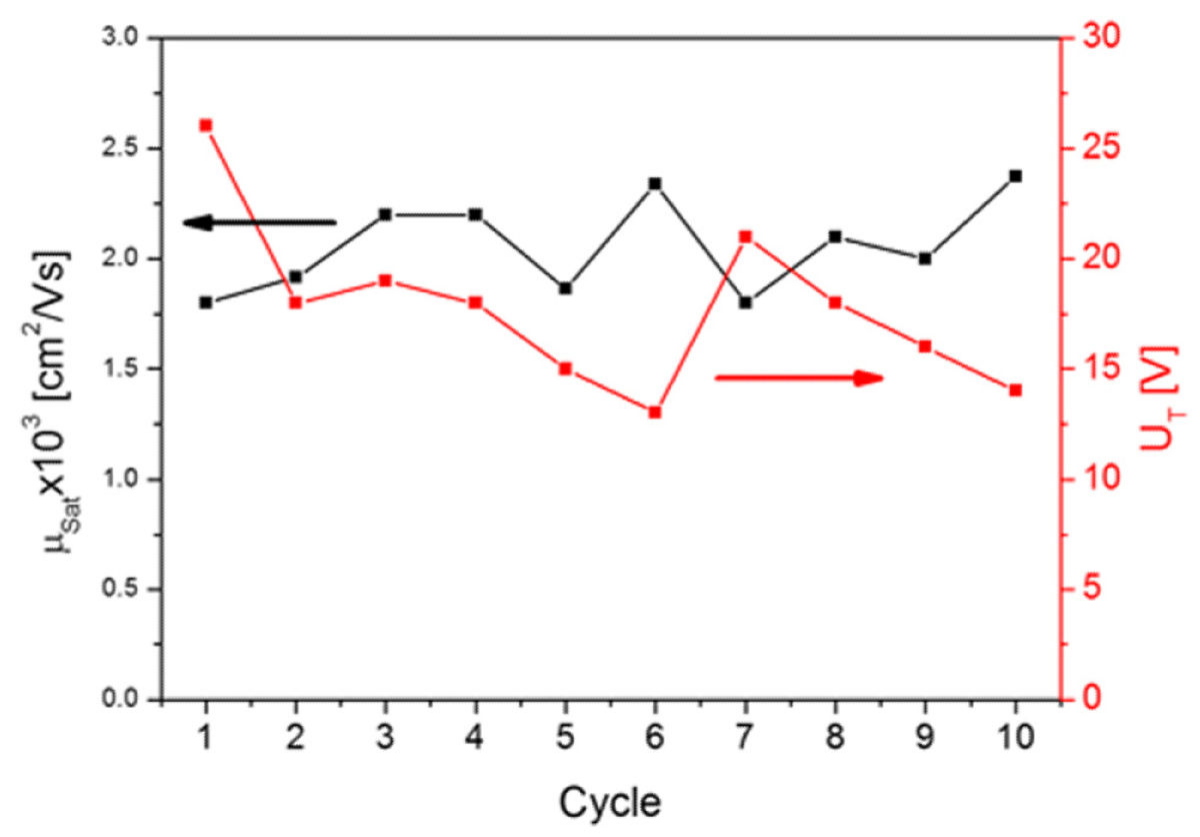
Saturation mobilities and threshold voltages for ten subsequently prepared devices by adsorption and desorption of 8 nm thick pentacene films. Adsorption and measurement temperature was 300 K.

Although the obtained mobilities are smaller than those reported in the literature for similar pentacene transistors in bottom contact configuration (0.1–0.3 cm^2^/Vs),^31^ the asset of our setup is that we can repeatedly desorb the pentacene film and deposit another layer of the same thickness, resulting in the same outcome within a relatively small error. This is shown in Fig. 4, where output and transfer characteristics were analyzed in terms of saturation mobility and threshold voltage for ten pentacene films with 8 nm thickness, prepared by repeated adsorption and desorption processes. The obtained values for the mobility do not take possible influences of the contact resistance into account. Thus, these values should be seen as effective mobilities, allowing a comparison in device-quality for specific preparation conditions. Actually, the particular non-linear shape of the output characteristic at low drain voltage (Fig. 3(a)) hints at an influence of the contact resistance.^30^

### C. Influence of sample preparation temperature

**FIG. 5:**
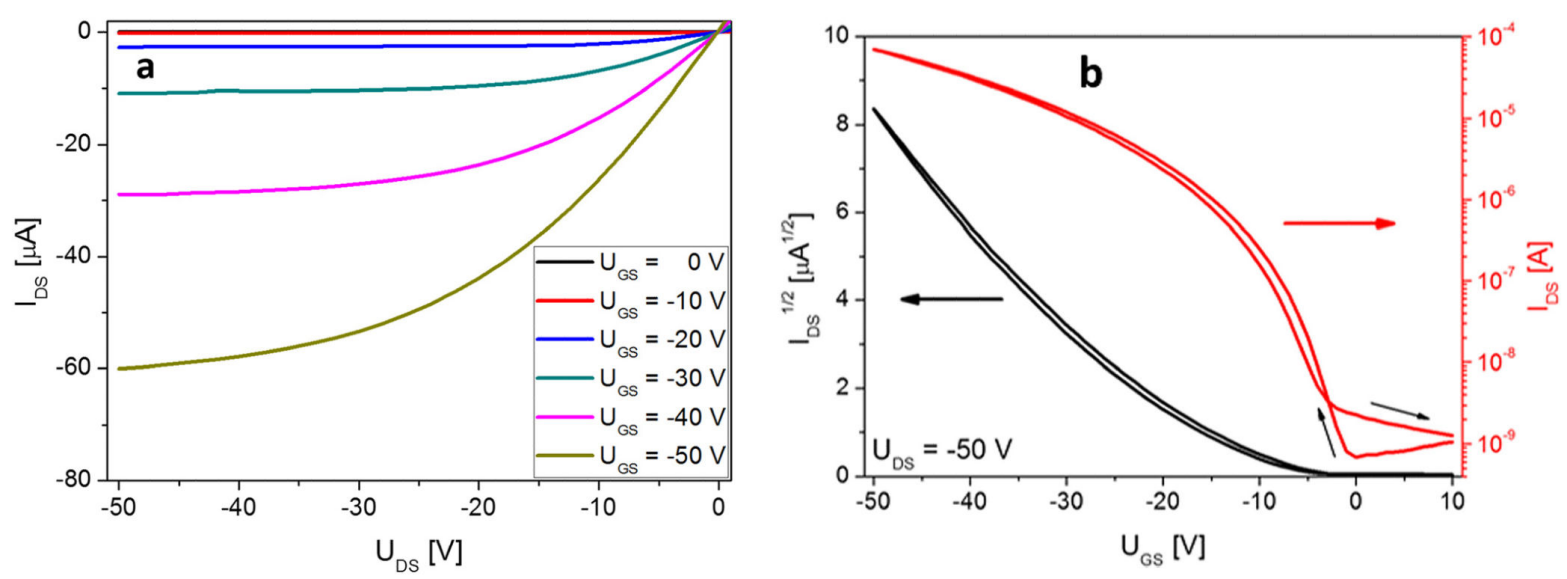
Output (a) and transfer characteristics (b), measured at 300 K, of an 8 nm thick pentacene film after deposition on a carbon covered device at 200 K.

**FIG. 6:**
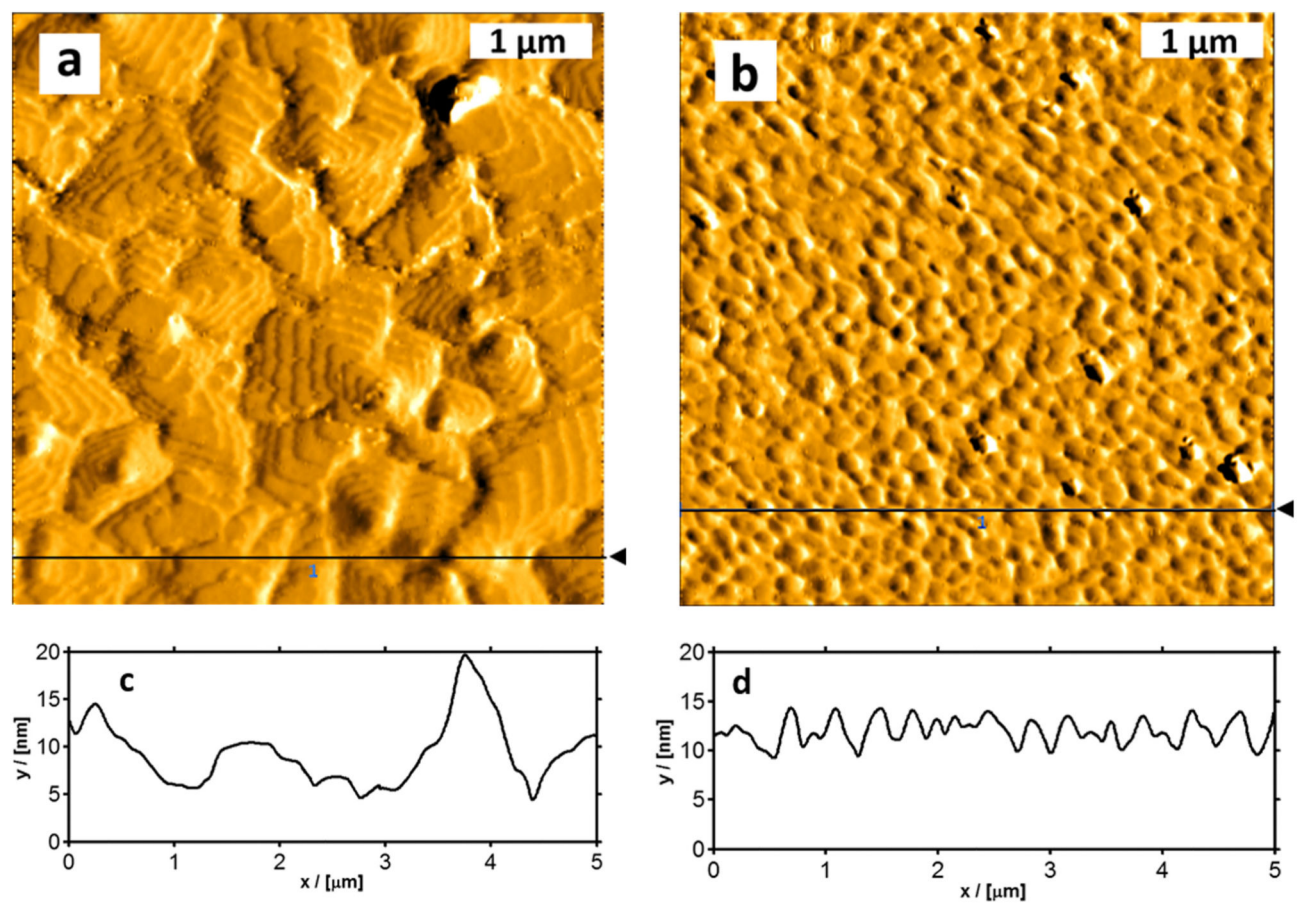
AFM images (5 *μ*m × 5 *μ*m) of 12 nm thick pentacene films on silicon dioxide, deposited at 300 K (a) and at 200 K (b), and the corresponding cross sections ((c) and (d)) along the line indicated in the AFM images. The measurements were performed at 300 K.

It is commonly known that device properties crucially depend on the morphology of the active layer in the channel and in the transition region between channel and contact area.^30^ In addition, the morphology depends on the substrate chemical composition, as well as on the deposition rate and substrate temperature, as described in some detail in the literature.^17,20,28^ In this section, we focus on the influence of the substrate temperature during deposition. Figs. 5(a) and 5(b) show output and transfer characteristics similar to Fig. 3, but in this case the 8 nm thick pentacene film was deposited at 200 K and then the device was warmed up to 300 K for electrical characterization. A significant improvement of the device performance could be observed. The mobilities increased by a factor of 15 (**μ*_Sat_*=3.6 × 10^−2^ cm^2^/Vs, **μ*_Lin_*=2.3 × 10^−2^ cm^2^/Vs), the on/off ratio is now ~10^5^, and *U_T_* and *U_on_* changed to −17 V and −1 V, respectively. It is most reasonable to assume that the change in the characteristics is due to morphological changes at the oxide/semiconductor interface and/or at the contact/semiconductor interface. This is supported by AFM measurements of 12nm thick pentacene films, deposited on SiO2 at 300 K and 200 K, respectively, as shown in Fig. 6. While the film deposited at 300 K shows the well-known terraced mounds of micrometer size,^17,32^ the film prepared at 200 K is composed of very small grains with an average size of less than 200 nm.

**FIG. 7:**
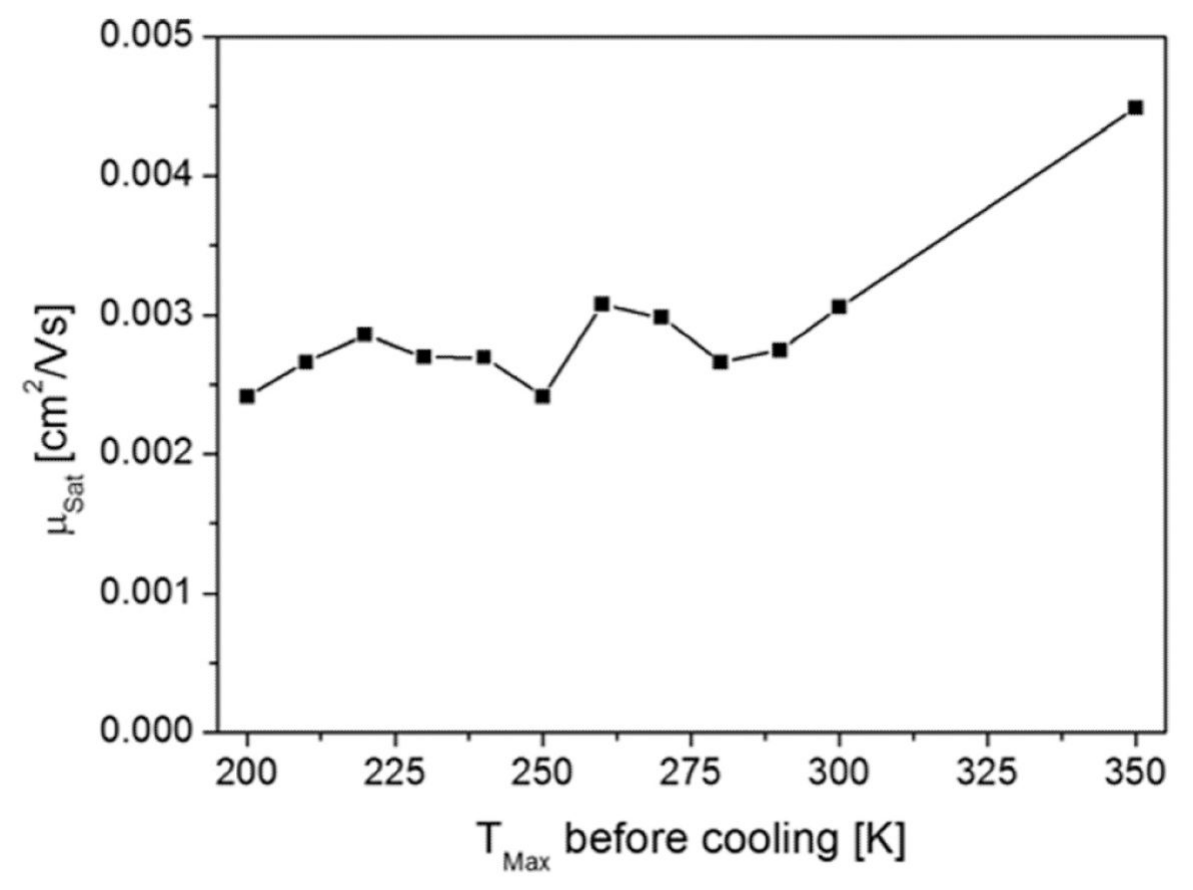
Improvement of the saturation mobility due to heating to the indicated temperatures of an 8 nm film deposited at 200 K. Device characterization was done at 200 K in all cases.

To get more information on this subject and to see if the morphological change happens at a particular temperature, we prepared an 8 nm thick film and repeatedly heated the device to increasing terminating temperatures and subsequently cooled to 200 K for electrical characterization. This is summarized in Fig. 7 where the saturation mobility is plotted vs the maximum heating temperature. For these experiments, the samples were heated with a rate of 1 K/s to the desired final temperature and immediately cooled down afterwards. This experiment shows that the performance of the device increases continuously, most probably due to a continuous irreversible morphological change of the active layer in the channel region and at the interface. For the value at 350 K, the film was held at this temperature for 5 min.

**FIG. 8:**
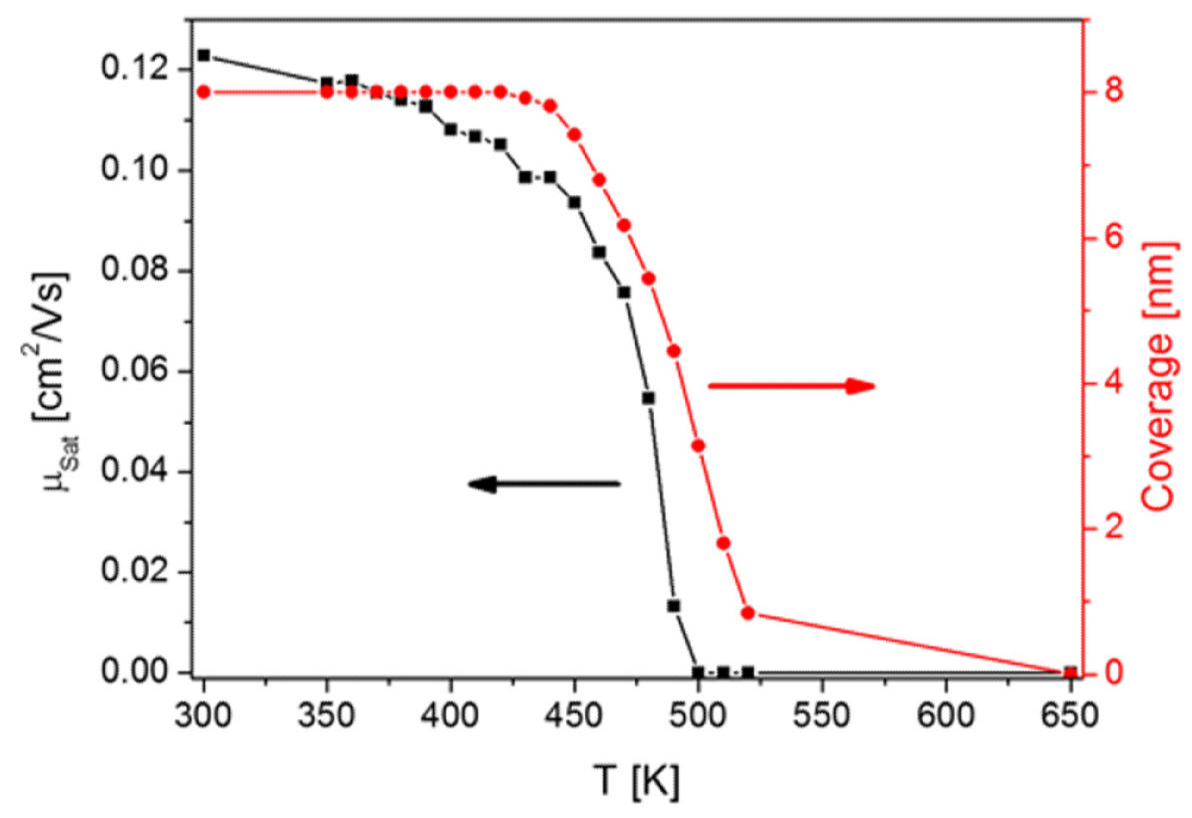
Mobility change with increasing annealing temperature up to the indicated temperature values after deposition of 8 nm pentacene at 200 K and initial annealing at 350 K for several hours. Device characterization was done at 300 K. In addition, the remaining coverage after each annealing step, as determined by TDS, is shown.

In order to check the thermal stability of the devices, similar experiments were performed by annealing films to increasing terminating temperatures up to the desorption temperature, as shown in Fig. 8. In this case, the pentacene film was deposited at 200 K and subsequently annealed at 350 K for several hours, before the actual experiment was started. The device characterization after the heating steps was always done at 300 K. This is the reason why in this case the mobilities are larger than those derived from Fig.5(b). It can be seen that already some diminution of the mobility sets in at an annealing temperature of about 450 K, although desorption of pentacene has not yet started. This hints to morphological changes in the channel area, most probably due to some dewetting processes. The mobility decreases drastically when desorption sets in, as determined by TDS, and already reaches zero with about 3 nm of pentacene still present on the surface. This is a further indication of strong dewetting prior and/or during desorption.

### D. Temperature dependence of the field effect mobility

**FIG. 9:**
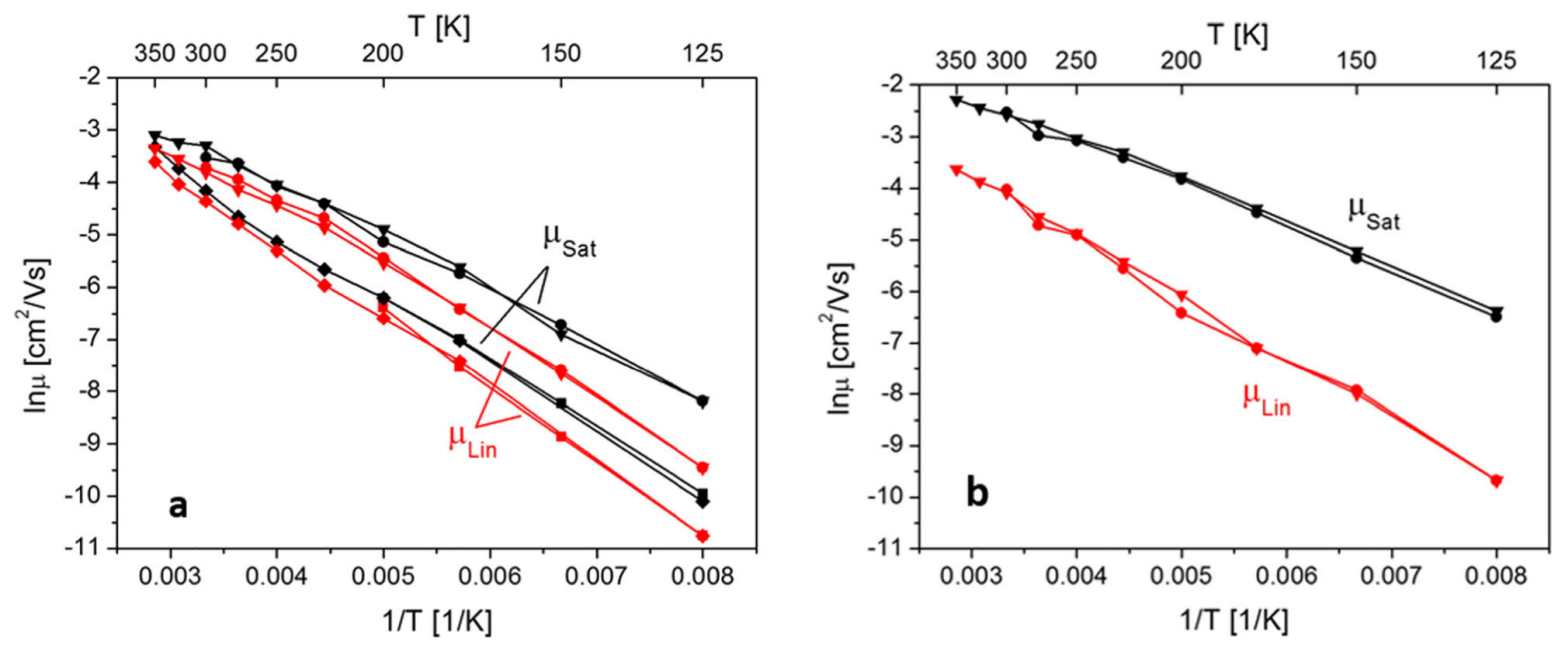
(a) Temperature dependence of saturation and linear mobility for an 80 nm thick pentacene device, prepared at 200 K, cooled to 125 K (squares), then heated to 350 K (diamonds), and subsequently cycled once more between 350 K and 125 K (circles and triangles). (b) Similar experiment after deposition of an 80 nm thick pentacene film at 300 K, cooled to 125 K (circles), and heated subsequently to 350 K (triangles).

After a pentacene film has been prepared at 200 K and annealed to 350 K, no further morphology changes can be expected when remaining in this temperature interval. This allows the measurement of the mobility as a function of device temperature and hence to get information on activation barriers involved in the generation of the drain current. In Fig. 9(a), the logarithm of the saturation mobility *μ*_sat_ and the linear mobility *μ*_lin_ are plotted vs 1/*T* for an 80 nm thick pentacene layer prepared at 200 K, then cooled to 125 K (squares), afterwards heated to 350 K (diamonds), and then again cycled between 350 K and 125 K (circles and triangles). One sees that the mobility is reversible between 200 K and 125 K, demonstrating that in this temperature range no morphological changes take place. However, increasing the temperature to 350 K leads to a stronger than expected increase of the mobility at around room temperature. A subsequent cooling/heating cycle between 350 K and 125 K does not show a hysteresis, indicating that the pentacene film is now morphologically stable in this temperature range. From the slope of these curves, one can deduce the involved activation barriers for the thermally activated carrier transport.

The meaning of the obtained activation barriers, however, is not immediately evident; it depends on the prevailing charge carrier mechanism. Several charge transport models have been discussed in the literature. Band-like charge transport is generally assumed not to play a significant role in polycrystalline or amorphous organic materials. A frequently discussed model is the so called MTR (multiple trapping and release) model, which assumes that most of the carriers are trapped in localized states, which have to be promoted temporarily into a delocalized band, in which charge transport occurs. In this case, the drain current depends on the activation barrier between the localized trap level and the delocalized band edge.^5,33^ In addition, in a polycrystalline film the charge transfer between the individual grains may also play an important role. If so, the measured activation barrier should be explained as the barrier necessary for thermionic emission of charge carriers between the individual pentacene grains.^34^ Under those circumstances, the mobility will strongly depend on the grain density of the film. Furthermore, the gold electrode may form a Schottky contact with the semiconductor, such that the thermally activated charge injection may dominate the temperature dependence of the drain current.

Due to the fact that our devices show a rather low mobility, attributable to the carbon contaminated interface, we assume that the contact resistance should play a minor role for the mobility determination from the transfer curves; the temperature dependence of the effective mobility should be largely determined by the properties of the organic film. According to the MTR model, the effective mobility is determined by a recurrent charge carrier trapping and release from shallow trap states below the conduction band edge. By assuming, for simplicity, a single trap state with an energy *E_A_* above the conduction band, the effective mobility can be described by

(3) $$\mu_{\mathrm{eff}}=\mu_{0}\alpha \cdot \exp\left(\frac{-E_{A}}{kT}\right),$$

where *μ*_0_ is the free carrier mobility near the bottom of the extended states and *α* = *N_c_*/*N_t_*, where *N_c_* denotes the density of states at the bottom of the band and *N_t_* the trap density.^5,32^ However, even in the case of non-single level trap states, e.g., in the case of a linear or uniform distribution of states, it has been shown that Eq. (3) still holds. The activation energy *E_A_* then corresponds to the difference between the lowest lying trap states and the extended band edge. The value of *α* depends of course on the distribution of the trap states, but can be approximated by *α* = *E_A_*/*kT*.^32^ Using this model, we can evaluate the data of Figs. 9(a) and 9(b). There we can see that the relationships ln *μ* vs 1/*T* do not follow a straight line, but are slightly bent, indicating a distribution of activation barriers. Nevertheless, a linear least squares fit through the data set of Fig. 9(a) (film prepared at 200 K) yields (mean) activation barriers of *E_A_* = 100 ± 10meV, obtained from the linear mobility (*U_GSmax_* = −50 V, *U_DS_* = −3 V) and *E_A_* = 91 ± 10meV, obtained from the saturation mobility (*U_GSmax_* = −50 V, *U_DS_* = −50 V). The absolute values for the saturation mobility are always larger by a factor of about 2 at room temperature than for the linear mobility and the difference increases to a factor of about 4 at 125K. Equivalent evaluation of the data set in Fig. 9(b) (film prepared at 300 K) yields activation energies of *E_A_* = 103 ± 10 meV, obtained from the linear mobility, and *E_A_* = 66 ± 10 meV as obtained from the saturation mobility. The derived values for the saturation mobilities are larger by a factor of 4 at 300 K and of about 30 at 125K compared to the linear mobilities.

For the pre-exponential factor *μ*_0_*α* of Eq. (3), measured at room temperature, we obtain the following values: For the 200 K prepared device, using **μ*_Lin_* yields *μ*_0_*α* = 1.15 cm^2^/Vs and using *μ_Sat_* yields *μ*_0_*α* = 1.08 cm^2^/Vs. For the 300 K prepared device, using *μ_Lin_* yields *μ*_0_*α* = 0.93 cm^2^/Vs and using **μ*_Sat_* yields *μ*_0_*α* = 1.03 cm^2^/Vs. It is interesting to note that we obtain similar values for *μ*_0_*α* (1.0 ± 0.1 cm^2^/Vs) for all cases, although the film prepared at 200 K shows a much higher grain density than the one prepared at 300 K (see Fig. 6). This suggests that the observed temperature dependence of the mobility is indeed mainly determined by the activation of shallow trap states in the pentacene islands, rather than by the thermionic emission between islands. Making use of the above mentioned relationship *α* = *E_A_*/*kT* and taking an average activation barrier of 80 meV into account, we obtain a value of *α* ≈ 3 , and consequently a free carrier mobility for pentacene of about 0.3 cm^2^/Vs.

**FIG. 10:**
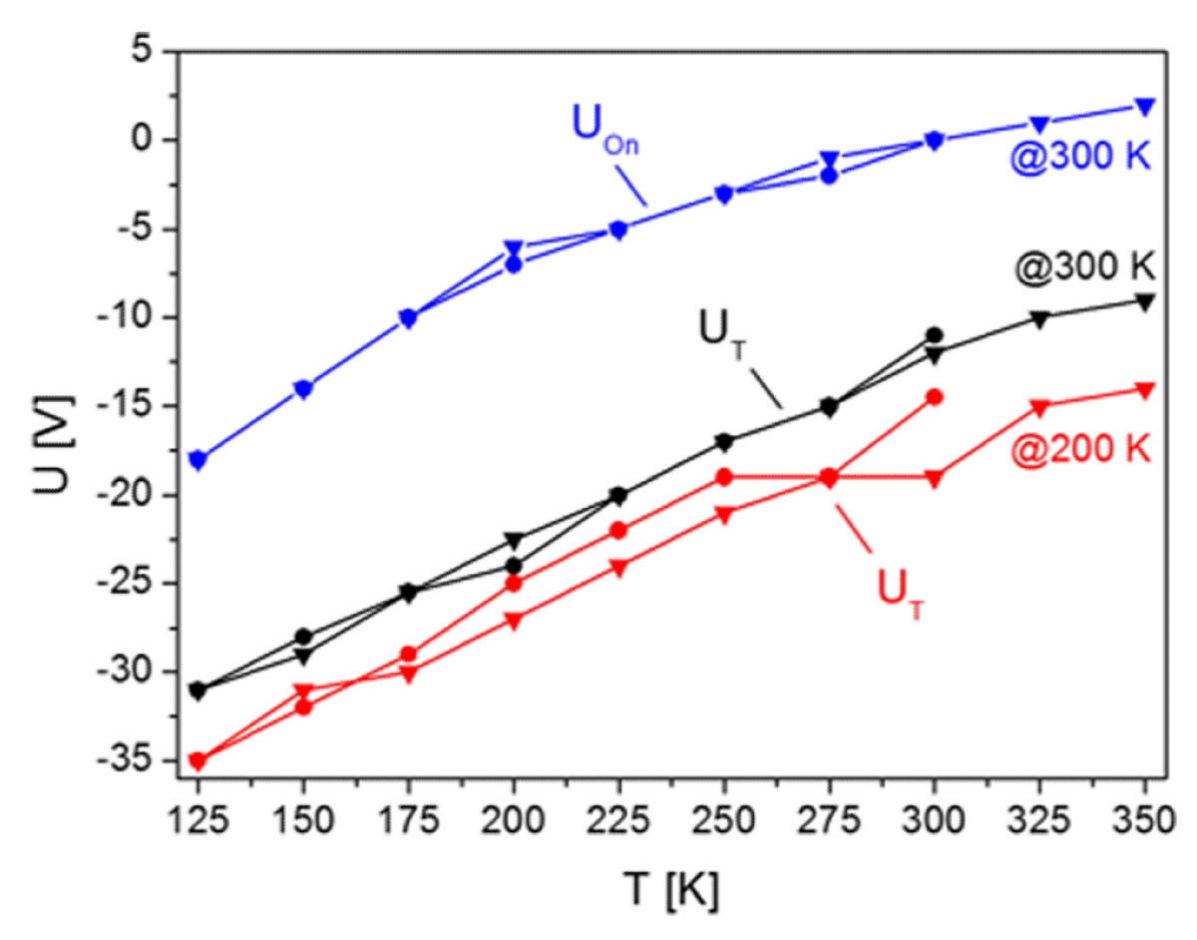
Temperature dependence of the threshold voltage *U_T_* and onset voltage *U*_on_ for 80 nm thick pentacene devices, prepared at 200 K and 300 K, respectively. *U*_on_ for the 200 K sample is, for clarity reasons, not shown, but has a similar offset compared to *U*_T_ , as shown for the 300 K sample.

Further information on the physics involved can be obtained from the changes of the threshold voltage *U_T_* and onset voltage *U_on_* as a function of temperature. These values are summarized in Fig. 10 for pentacene films prepared at 200 K and 300 K, respectively. The threshold voltage increases nearly linearly from about −35 V at 150 K to about −14 V at 350 K for the film prepared at 200 K, and from −31 V at 150 K to −9 V at 350 K, for the film prepared at 300 K. Similarly, the onset voltage changes from −18 V at 125 K to +2 V at 350 K for the 300 K sample. For the 200 K sample, *U_on_* changes from −20 V to +4 V in the same temperature regime (not shown for clarity reasons). Both parameters, *U_T_* and Uon, have the same origin and have similar meaning, and they are just different due to different evaluation procedures. The threshold voltage, which shows up in Eqs. (1) and (2), can be seen as the gate-source voltage required to obtain *appreciable* drain current.^10^

The increase of the threshold voltage (higher negative values) with decreasing temperature can be explained by the mobility edge model.^35^ In this model, two types of carriers are classified: mobile carriers in the band-like state and immobile carriers in the trap states. The trap states extend more or less (deep and shallow traps) into the gap in an exponential like form. For hole conductivity, the density of occupied states *D(E)* is given by

(4) $$N=\int D(E)f(E,E_{F})dE,$$

where *f*(*E,E_F_*) is the Fermi-Dirac distribution for holes. By increasing the (negative) gate voltage, the Fermi level moves towards the hole conduction band and the deep traps become more and more filled until at a certain gate voltage (the threshold voltage) states in the delocalized band and shallow trap states become filled as well and charge starts to flow in the channel. At lower temperature, the tail of the Fermi-Dirac distribution is less pronounced, thus a higher (negative) gate voltage is needed to fill the traps. The fact that for the devices prepared at 200 K a higher negative threshold voltage is needed, compared to the devices prepared at 300 K, hints to a correlation between the density of deep traps and the grain density in the semiconducting film (see Fig. 6). Thus, one can draw the conclusion that the deep traps are mainly located at the grain boundaries.

The threshold voltage, as observed for disordered organic field-effect transistors, is seen as a mere fit parameter by some authors, lacking a clear physical basis.^36^ As a characterization parameter these authors suggest the onset voltage to be used, which is defined as the flat-band voltage. According to their calculations, the onset voltage should be close to zero and in particular independent of the temperature. This is apparently not the case in our work, where the onset voltage shows a shift with temperature comparable to the threshold voltage.

### E. Thickness dependence of the mobility

A further advantage of *in situ* film preparation and simultaneous electrical characterization is the possibility to determine the electrical behavior as a function of film thickness. There exists some literature dealing with this subject for pentacene devices; however, the reported results show quite a few discrepancies. While there is agreement that drain-source current starts to flow when the first monolayer is completed (or slightly before), there are differing statements as to the saturation of the drain current (or mobility) and the change of the threshold voltage with film thickness. Park *et al*.^37^ reported a drain current saturation for pentacene at two monolayers (3 nm) already, Liu *et al*.^17^ observed saturation around 7 nm, Kiguchi *et al*.,^16^ Shehu *et al*.,^20^ and Ruiz *et al*.^38^ found saturation at around 10 nm, Fiebig *et al*.^22^ noted saturation of the mobility above 30 nm and Wang and Cheng^39^ reported threshold voltage (and mobility) changes up to 180 nm.

**FIG. 11:**
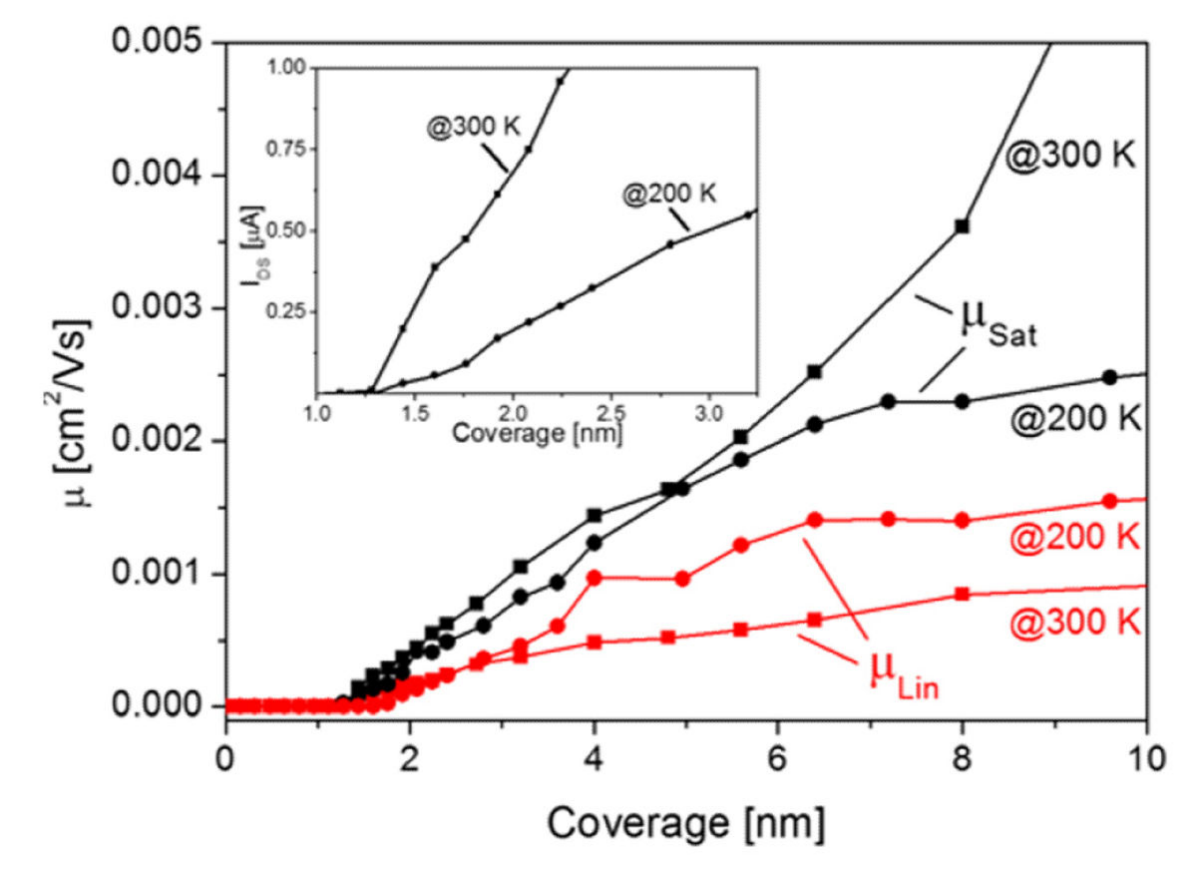
Linear (red symbols) and saturation (black symbols) mobilities in the low coverage regime for pentacene films prepared and measured at 200 K (circles) and 300 K (squares), respectively. In the inset, the onset of *I_DS_* in the very low coverage regime is shown.

In our experiments, we have measured output and transfer characteristics as a function of thickness for devices prepared at 300 K and 200 K and evaluated the linear and saturation mobilities, as well as the threshold and onset voltages. In Fig. 11, **μ*_Lin_* and **μ*_Sat_* are compiled for films in the range of 0–10 nm mean thickness.

**FIG. 12:**
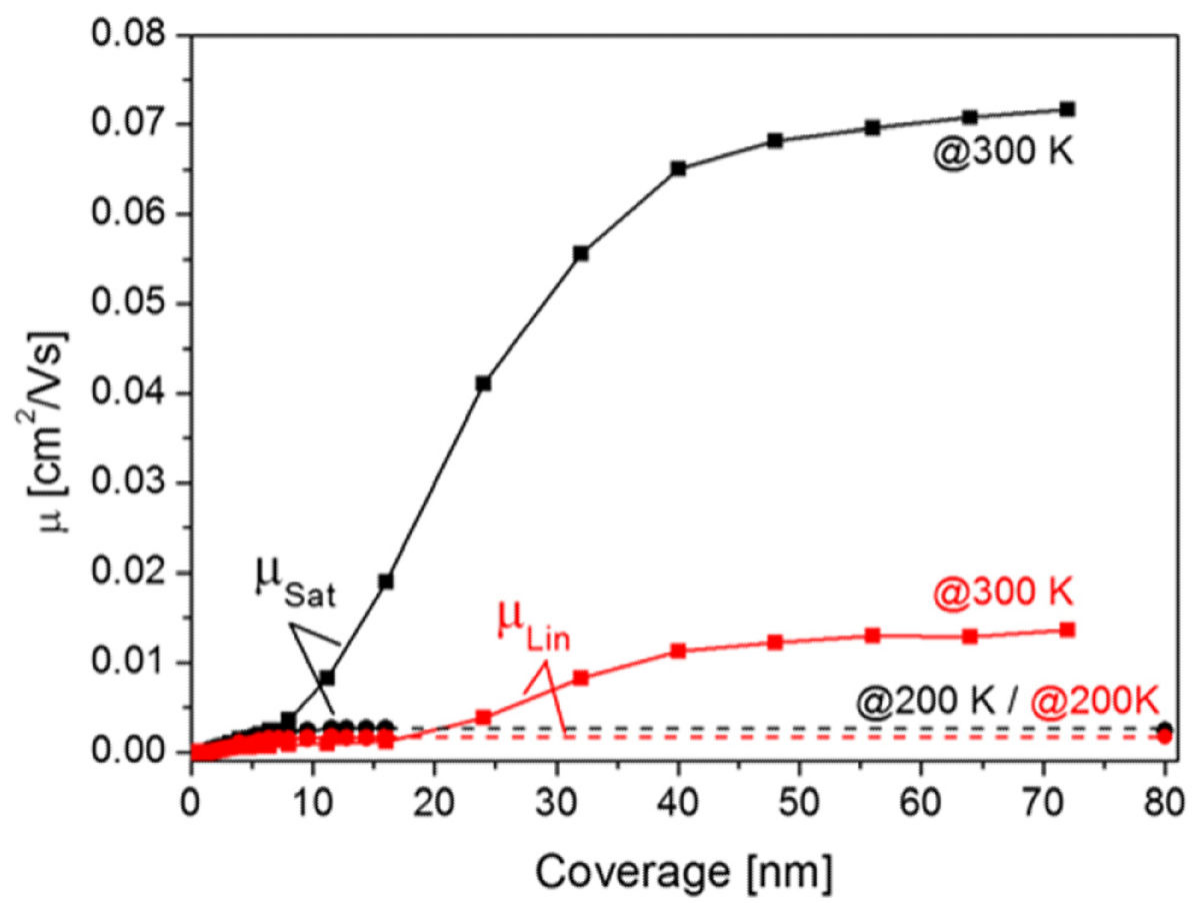
Linear (red symbols) and saturation (black symbols) mobilities in the high coverage regime for pentacene films prepared and measured at 200 K (circles) and 300 K (squares).

**FIG. 13:**
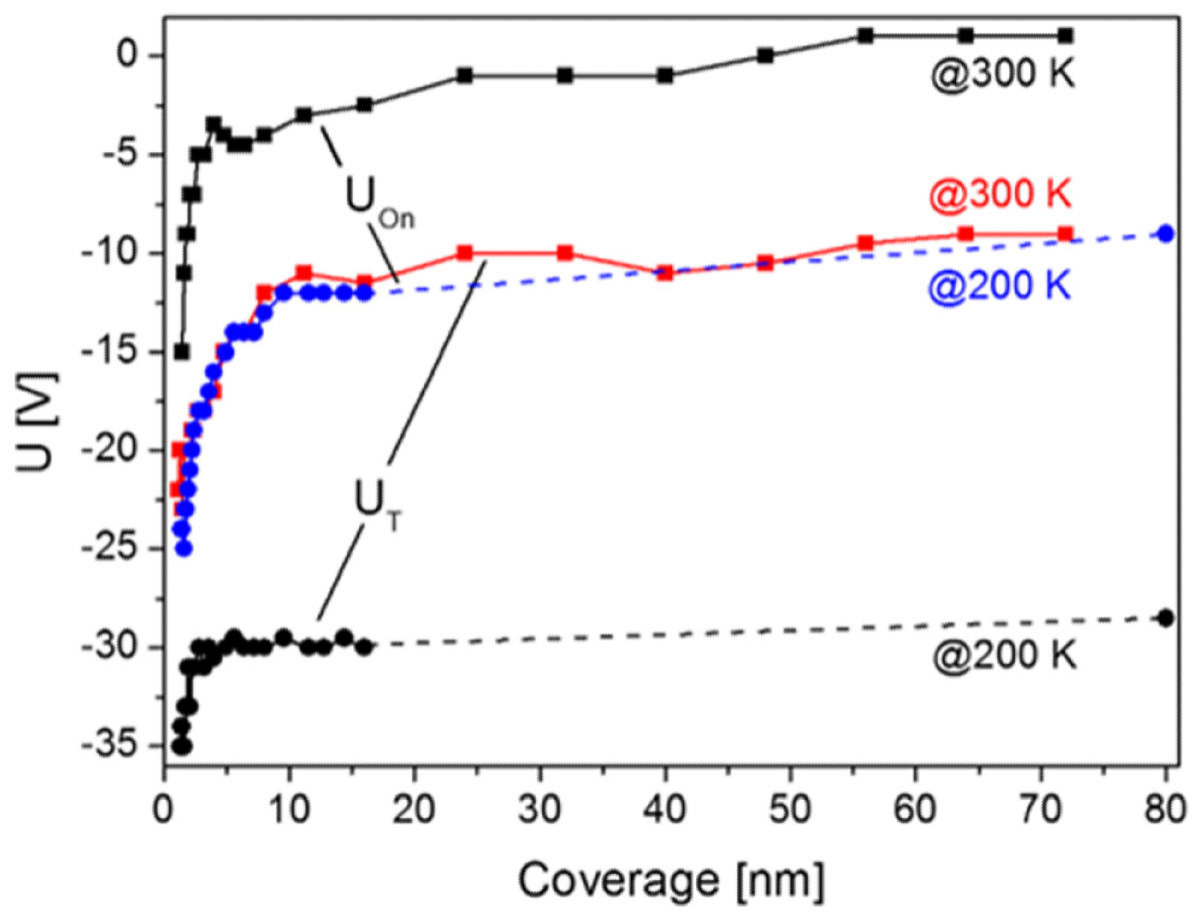
Coverage dependence of the threshold and onset voltage for pentacene devices prepared at 200 K and 300 K.

In the inset of Fig. 11, the onset of the drain current at the very beginning of channel conductivity is shown. One observes a sharp onset at about 1.3 nm, equivalent to a coverage of 0.86 monolayers of standing molecules. This agrees quite well with the percolation threshold of about 0.7 monolayers, depending on the models used.^40-42^ The fact that the mobility is smaller for the film when prepared and measured at 200 K is mainly due to its temperature dependence, as shown in the previous chapter. With increasing coverage, the linear mobility increases and reaches a weakly pronounced saturation at about 6 nm (4 ML) for both the 200 K and 300 K prepared films. Interestingly, the saturation mobility for a film prepared at room temperature does not show a saturation in this coverage range. In Fig. 12, the evolution of the mobilities for even thicker films is depicted. For the film deposited at 200 K, a final saturation of the mobilities is reached at about 10 nm. However, for the film prepared at room temperature a further mobility increase is observed, which does not show saturation before a coverage of about 40 nm. Considering the fact that the mobilities measured at 300 K are by about a factor of 5 larger than those measured at 200 K (for the same morphology), as shown in the previous chapter, it is interesting to note that the saturation mobility for the thick film prepared at 300 K is still larger than for the film prepared at 200 K, but measured at 300 K. However, the opposite behavior is observed in the low coverage regime (<10 nm). Here, the temperature corrected *μ_Lin_* and *μ_Sat_* are significantly larger for the films prepared at 200 K compared to those prepared at 300 K. For completeness, we have also evaluated the threshold and onset voltage as a function of pentacene coverage, prepared at 200 K and 300 K, respectively, as shown in Fig. 13.

From this set of data, we can draw the following conclusions: The onset of the channel current agrees quite well with the percolation of the first monolayer of standing pentacene molecules, more or less independent of the substrate temperature. With increasing coverage, the measured mobilities increase and reach a first saturation at about 6 nm (4 ML). Taking the temperature dependence of the mobility into account, we see that in this coverage regime the mobility for 200 K prepared films is by a factor of about 5 higher than that for 300 K devices. This is most likely a result of the different film morphologies. At room temperature, pentacene shows clear Stranski-Krastanov growth,^43^ where the first layer nearly fully closes before further layers start to form. The additional layers then show strong islanding, due to a high Ehrlich-Schwöbel barrier^44,45^ for step-down diffusion (see Fig. 6). At 200 K sample temperature, the first layer still develops quite well, although the island density is much higher. However, the following layers are also composed of many small islands, where step-down diffusion is facilitated and a more layer-like film growth takes place. According to the investigations of the Biscarini group,^20^ it is known that not only the first layer contributes to the channel current but also that the effective Debye length can reach up to 5 ML into the semiconductor. Thus, in a fine-grained, layer-like film, several layers, which are already percolated, will allow more charge to flow than in a Stranski-Krastanov film, where only little percolation exists above the first layer. On the other hand, at higher grain density more charge traps will be generated, which decreases the mobility.^46^ Thus, at higher coverage when finally many layers of the 300 K prepared film are percolated, the smaller grain density and probably also the better molecular structure in the grains will finally yield a higher mobility for the 300 K film than for the pentacene film prepared at 200 K.

In addition to the grain density and percolation within the film, the contact between the semiconductor and the gold electrodes (access resistance) will finally determine the channel current and thus the effective mobility. We believe that this is the reason for the further increase in mobility above 15 nm for the 300 K film, which finally leads to saturation above 50 nm mean thickness. This assumption is supported by the coverage dependence of the threshold and onset voltage, which are characteristic quantities reflecting the physics involved in the charge carrier transport. We observe a significant change of these values only up to a coverage of about 10 nm, for the 300 K film, and up to about 5 nm for the 200 K film. This is the coverage regime where the mobility is governed by increasing film thickness within the Debye length. For the 200 K film, the initial *U*_T_ has a value of −35 V, whereas *U*_T_ = −23 V for the 300 K film. This is in accord with the above stated correlation between the threshold voltage and the density of deep traps due to the grain density. Interestingly, with increasing coverage the threshold voltage changes to less negative values by about 13 V for the 300 K film and by about 5 V for the 200 K film, which would suggest a decrease of the deep hole traps with increasing film thickness. Fiebig *et al*.,^22^ who found a very similar threshold change as a function of coverage, postulated the formation of electron traps in the semiconductor and/or the surface of the film. With increasing film thickness more electron traps exist, which are filled by electrons. This leads to negative charging of the film and consequently a more positive gate voltage is needed for compensation. For the 200 K film, the surface is less corrugated and a constant threshold voltage is reached earlier than for the 300 K film, which is composed of large mounds. The fact that no significant change of the threshold and onset voltage with further coverage increase takes place demonstrates that the mobility change in this coverage range is not due to a conductivity change in the semiconductor but most likely due to a changed access resistance at the Au-pentacene interface.

## IV. SUMMARY AND CONCLUSIONS

In this work, we have performed *in situ* preparation, electrical and surface analytical characterisation of bottomgate, bottom-contact pentacene transistors, which allowed the fabrication of highly reproducible devices. With a special sample holder setup, we were able to cool any device to 125 K and heat it to 800 K, if so desired. By repeated deposition and desorption of pentacene on the model device, both the gold contact pads and the SiO_2_ channel were intentionally contaminated with carbon, as verified by Auger electron spectroscopy. Although this surface modification reduced the absolute values of the charge carrier mobility, we could repeatedly establish devices with unprecedented reproducibility. We focused on the impact of the film morphology on the temperature and coverage dependency of the mobility and threshold voltage. While the deposition of pentacene at room temperature leads to the well-known Stranski-Krastanov film morphology, deposition at 200 K results in a fine grained, nearly layer like film. We were able to show that the threshold voltage is mainly governed by deep traps induced by the grain boundaries, while the temperature dependence of the mobility can be well described by the multiple trapping and release model, when assuming that the shallow traps are mainly located within the grains. With respect to the coverage dependency, we see the onset of channel conductance at a percolation threshold of about 0.8 ML, followed by an intermediate saturation of the mobility at around 4 ML. This demonstrates that charge transport is not restricted to the oxide/semiconductor interface and the very first layer, as sometimes assumed, but that rather several layers contribute to the channel current. In this coverage range, the mobility for the 200 K prepared device is larger than that for the 300 K device, due to the more layer-like film morphology within the Debye length. With further coverage increase, the mobility of the 300 K device once more increases until a saturation is eventually reached at about 50 nm thickness. Since this continued mobility increase does not take place for the 200 K device we trace this behaviour back to insufficient connection of the pentacene film to the gold contacts at higher substrate temperature due to poor wetting (increased access resistance). For a sufficiently thick film, when charge transport is not limited by the contact resistance, films grown at 300 K show higher mobilities than those deposited at 200 K sample temperature, due to the smaller grain density and probably also due to a better molecular structure within the grains.

## Supplementary Material

Supporting Information
