# Supplementary material for: In situ preparation, electrical and surface analytical characterization of pentacene thin film transistors
Source: J Appl Phys. Author manuscript; Available in PMC 2015 Mar 24. (PMC4371883; doi:10.1063/1.4895992)
Supplement: Supporting Information [file NIHMS62563-supplement-1.pdf]

Supporting Information

In-situ preparation, electrical and surface analytical characterization of pentacene thin film transistors

R. Lassnig[†], *B. Striedinger**, *M. Hollerer* [†], *A. Fian**, *B. Stadlober** *and A. Winkler*[†,a)]

† Institute of Solid State Physics, Graz University of Technology, Petersgasse 16, A-8010 Graz, Austria

* Materials Division, Joanneum Research Materials, Franz-Pichler-Straße 30, A-8160 Weiz, Austria

SI-1. Thermal desorption of pentacene from the stainless steel sample holder

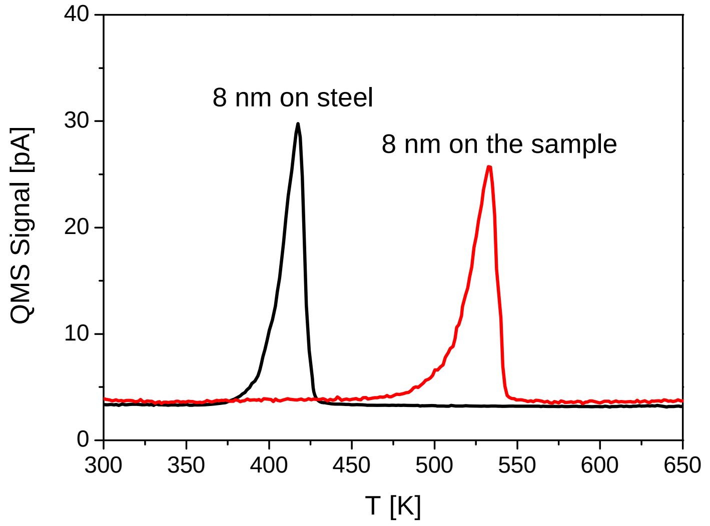

FIG. SI-1. Comparison between thermal desorption spectra of pentacene from the stainless steel sample holder and a standard sample surface. Nominal layer thicknesses was determined by a quartz microbalance. Adsorption temperature T_{ad} = 300 K, heating rate β = 1 K/s. The peak maximum for the steel plate desorption is reached at 420 K.

SI-2. Temperature correction for the Au/SiO_2 sample surface on the stainless steel sample holder

Due to the special mounting setup and thermal resistance between the heated steel plate and the silicon wafer the temperature on the sample surface lags behind the temperature measured by the thermocouple. From figure SI-1 we know, that the 8 nm thick multilayer (formed by an exposure equivalent to a 100 Hz resonance frequency change for the quartz microbalance) desorbs at 420 K. A pentacene layer of the same nominal layer thickness on the carbon covered Au/SiO_2 sample surface shows a desorption peak at 530 K, which in turn again corresponds to the true desorption temperature of 420 K. Thus we can correct the temperature scale by assuming that during adsorption both the steel plate and the SiO_2 substrate share the same temperature, and that the temperature lag during the heating process follows a linear dependence. The correction formula can then be expressed as

$$T_{corr} = T_0 + \left(\frac{T_p^{SS} - T_0}{T_p^{Si} - T_0}\right)(T - T_{0)}$$

with the adsorption temperature T_0 (300 K), the desorption temperature for a multilayer from the steel plate T_p^{SS} (420 K) and the desorption temperature for a multilayer from the Au/SiO_2 silicon wafer surface T_p^{Si} (530 K).
